# Functional Synergies Underlying Control of Upright Posture during Changes in Head Orientation

**DOI:** 10.1371/journal.pone.0041583

**Published:** 2012-08-01

**Authors:** Eunse Park, Gregor Schöner, John P. Scholz

**Affiliations:** 1 Biomechanics and Movement Science Program, University of Delaware, Newark, Delaware, United States of America; 2 Institute für Neuroinformatik, Ruhr University, Bochum, Germany; 3 Physical Therapy Department, University of Delaware, Newark, Delaware, United States of America; McMaster University, Canada

## Abstract

**Background:**

Studies of human upright posture typically have stressed the need to control ankle and hip joints to achieve postural stability. Recent studies, however, suggest that postural stability involves multi degree-of-freedom (DOF) coordination, especially when performing supra-postural tasks. This study investigated kinematic synergies related to control of the body’s position in space (two, four and six DOF models) and changes in the head’s orientation (six DOF model).

**Methodology/Principal Findings:**

Subjects either tracked a vertically moving target with a head-mounted laser pointer or fixated a stationary point during 4-min trials. Uncontrolled manifold (UCM) analysis was performed across tracking cycles at each point in time to determine the structure of joint configuration variance related to postural stability or tracking consistency. The effect of simulated removal of covariance among joints on that structure was investigated to further determine the role of multijoint coordination. Results indicated that cervical joint motion was poorly coordinated with other joints to stabilize the position of the body center of mass (CM). However, cervical joints were coordinated in a flexible manner with more caudal joints to achieve consistent changes in head orientation.

**Conclusions/Significance:**

An understanding of multijoint coordination requires reference to the stability/control of important performance variables. The nature of that coordination differs depending on the reference variable. Stability of upright posture primarily involved multijoint coordination of lower extremity and lower trunk joints. Consistent changes in the orientation of the head, however, required flexible coordination of those joints with motion of the cervical spine. A two-segment model of postural control was unable to account for the observed stability of the CM position during the tracking task, further supporting the need to consider multijoint coordination to understand postural stability.

## Introduction

Except perhaps for the Queen’s Guard at Buckingham Palace or similar sentry-like occupations, humans rarely stand upright without performing other tasks. Even if the arms and body are generally quiet, individuals often track objects with their head, for example, a ball in flight before reaching up to catch it, or simply watching the flight of a shooting star. For an individual weighing 80-kg, the approximately 6.3-kg weight of the head is not insubstantial [1]. Thus, directional changes in the head’s orientation, especially if relatively rapid, have the potential to disturb posture and may require anticipatory postural adjustments elsewhere. This will depend, of course, on how closely head-neck motion is linked to that of the rest of the body. On the other hand, if head-neck DOFs were largely decoupled from body movement, then postural adjustments to head movement might be unnecessary.

Assuming that postural adjustments are required when tracking objects with the head, it is of interest to know whether they involve all joints along the kinematic chain or only ankle and hip joint movements. Generally, standing quietly is presumed to require primarily active control of ankle joint motion [2]–[4], with additional coordination with hip motion when stronger environmental and/or task constraints are imposed [5]–[8]. There have been a limited number of studies of how head motion influences muscle and/or joint coordination related to postural control [9]–[11]. Those studies did not distinguish between coordination related to maintaining upright posture versus that required for changing the head’s orientation. Instead, the focus was on the time course of changes in postural coordination of only the ankle and hip joints, indexed by the relative phase of their motions, as task and environmental constraints were varied. Whether a single or double inverted pendulum model of postural control is adequate to account for many aspects of upright stability is, nonetheless, an open question, particularly when additional tasks are performed in standing. Indeed, other joints along the body axis have been shown to be just as active as the ankle and hip joints even during quiet standing [12].

Movement of the head when performing tracking or orienting movements leads to transformations of sensory information from visual and vestibular receptors as well as their integration with changes in neck proprioceptor signals [13], [14]. Such sensory transformations are unlikely to have a strong effect on posture unless sensory conflict arises because the nervous system is believed to interpret such information via efference copy related to the motor commands generated to produce the head movement (Although alternative explanations have been offered [15]). Nevertheless, this process is not perfect [16], [17] so that the transformations of visual, vestibular and neck proprioceptive information during the tracking task could have subtle effects on posture beyond any mechanical coupling of neck DOFs with caudal body segments.

Accurate tracking of an external object when eye movement alone is inadequate would require, at the very least, adequate control of cervical joint motion. In principle, the task could be accomplished by independent control of cervical movements, including the atlanto-occipital (AO) joint. This would require that the excursion of more caudal joints of the body be of limited extent. Ankle joint movement, for example, has the potential to affect the head’s spatial orientation given its long lever arm with respect to the head’s position. Whether movement of other joints significantly affects the tracking task will depend, of course, on the required precision of that task. Thus, the need to coordinate caudal joints that are more related to postural control with those primarily involved in producing the desired changes in head orientation would arise only when the typically small postural sway exceeds the required precision of the task. For example, a 1.8-m shooter aiming at a 10-cm diameter target at a distance of 100-m who sways more than 0.05 degrees about the ankle risks missing the target unless she coordinates posture with the act of aiming and shooting at the target. Fortunately, normal postural sway is of relatively small amplitude, so the effect of postural sway on head orientation might be inconsequential if the precision requirements of tracking are not too great.

The purposes of this study, then, were to investigate multijoint coordination related to both stabilization of upright posture and the control of head orientation when having to track a visual target. Specifically, we asked whether the coordination of cervical joint motion with other joints of the body was necessary to stabilize upright posture during performance of the tracking task or was primarily related to producing consistent changes in the head’s orientation required for accurate tracking. To be clear, if cervical DOFs have little effect on the anterior-posterior (AP) motion of the whole body, then changes in the head’s orientation required to track a moving target could be achieved without related postural adjustments to stabilize upright posture. If so, movements of cervical joints should have weak indices of coordination with more caudal joints that are most related to postural control. However, to the extent that changes in head orientation affect the body posture, cervical joint motions should be more strongly coordinated with those of other joints. Moreover, if natural postural sway were within the precision requirements of the tracking task, then coordination of cervical motion with other joints should be unnecessary to accomplish tracking. However, the amount of sway about the ankle observed in the current study, combined with the target distance and size, indicated that it would affect targeting accuracy if postural changes were not coordinated with cervical movement. Thus, we expected to find strong indices of coordination between cervical and more caudal joint motion related to control of head orientation but weaker indices of coordination related to the stability of the body’s position in space. Most indices of coordination, e.g., cross-correlation analysis, relative phase analysis, non-negative matrix factorization or principal components analysis, cannot easily differentiate between these possibilities, however. For example, if such measures were to show a high correlation between the motions of cervical joints and joints more directly related to the control of body posture, it would be impossible to tell directly what was the goal of that coordination, i.e., to stabilize posture, change the orientation of the head in space, or a combination of both. Analysis based on the Uncontrolled Manifold (UCM) approach, however, provides a framework for addressing such questions because it is possible to determine how the variance of joint motions relates to changes in the value of variables more directly related to a task, such as consistency of head orientation or postural stability. This method was applied to experimentally obtained data in the current study, combined with UCM analysis of data with covariation among the actual joint motions artificially removed to simulate incoordination [18], [19]. That analysis helped to determine the extent to which UCM effects were primarily due to geometric constraints (i.e., variance of a variable that lies parallel to a dimension of the UCM and, therefore, cannot affect the task variable of interest) versus interjoint coordination.

## Methods

### Ethics Statement

The University of Delaware IRB approved the research described in this submission. Approval was based on an appropriate risk/benefit ratio and a study design wherein the risks were minimized. All research was conducted in accordance with the approved protocol. This protocol received expedited review based on the applicable federal regulation. Informed consent is a process beginning with a description of the study and insurance of participant understanding followed by a signed consent form. Informed consent continued throughout the study via a dialogue between the researcher and research participants. In accordance with Federal regulations, each participant received a copy of the signed consent document.

### Subjects

Twelve subjects (24.4±5.05 years old; 5 females and 7 males) volunteered and were paid to participate in this study in response to an online solicitation. Subjects had no balance disorders or dizziness, and no musculoskeletal injuries, neurological disorders or uncorrected visual acuity deficits by self-report. All subjects provided written informed consent according to the procedures approved by the Institutional Review Board of the University of Delaware consistent with the Declaration of Helsinki.

### Experimental Setup

Two stationary, 10-cm diameter circular targets were projected on a wall 2-m in front of the subject. The upper circular target was placed at eye level and the lower circular target was placed at 70% of eye level, measured from the floor. One 5-cm diameter, yellow circular cursor also was projected and oscillated sinusoidally between the two stationary circular targets at a frequency of 0.258-Hz. This frequency was chosen based on pilot experiments with three subjects that attempted to identify a frequency of tracking with which subjects could be successful while still being challenged.

**Figure 1:**
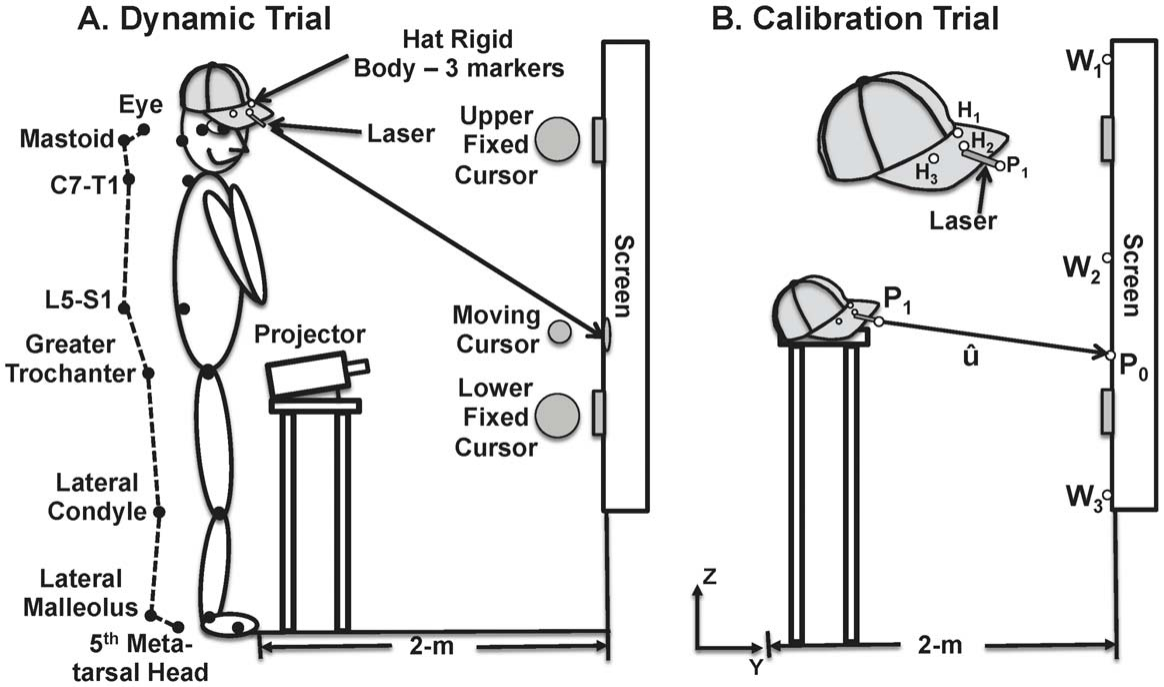
Experimental setup. A. Cartoon of experimental setup showing link-segment model, reflective marker locations, and position of subject relative to the projection screen mounted on a rigid wall. Circles indicate frontal plane view of 10-cm diameter fixed targets and 5-cm diameter moving target; B. Calibration trial had the hat placed on a high stool. H_1_, H_2_, and H_3_ are the reflective markers on the hat brim comprising the rigid body from which a local coordinate system was constructed. P_1_ is the reflective marker placed during calibration at the location where the beam was emitted from the laser. P_0_ is the point of intersection of the laser with the screen, calibrated with a reflective marker. P_1_ and P_0_ were used to determine the unit vector û along the laser beam in relation to the local coordinate system of the hat rigid body. Wall markers W_1_, W_2_, and W_3_ were used to calibrate the wall’s position in the global coordinate frame.

Experimental data were collected at a sampling rate of 120-Hz with a Vicon™ MX-13 (Oxford Metrics) motion-measurement system composed of eight infrared cameras. All analyses were performed in the sagittal plane. Reflective markers were placed at approximate joint centers on the right side of the body and on the brim of a baseball cap worn by the subject (Figure 1) to track body segment movement.

### Experimental Procedure

Subjects were required to stand barefoot on the floor with their feet shoulder-width apart, arms folded across their chest, for each trial. Subjects’ foot positions were marked to maintain the same position across trials. The laser pointer used to track the moving target was attached securely to the brim of a snuggly-fitting baseball cap that subjects wore.

Each subject performed three 4-min trials each for two conditions: (1) quiet standing while fixing their gaze at the center of two stationary targets; (2) standing in place while tracking a moving cursor between the two fixed targets with the laser mounted on the cap that they wore. To avoid biasing the subject’s preferred movement strategy, it was not specified how they should move their body to track the target. However, all subjects used their head rather than trunk movement to track the moving target. Subjects were provided rest as much as they wanted between trials. Trials of both tasks were randomized for each subject.

### Data Reduction

The three dimensional (3D) positions of the reflective markers were reconstructed with NEXUS (VICON™) software. The position information of each marker was filtered in Matlab™ using 4^th^ order Butterworth low-pass recursive filter with a 5-Hz cut-off frequency.

The projection of the laser’s beam onto the wall at each moment in time was estimated geometrically from data obtained during a static calibration trial. Three reflective markers (H_1_, H_2_, H_3_) were placed in a fixed position on the brim of the hat that subjects wore during the experiment to form a rigid body (Fig. 1). Also fixed rigidly to the hat’s brim was the laser pointer. During a static calibration trial (Fig. 1B), the hat was placed on a high stool such that it pointed toward the target screen, which was mounted on the wall in front of the subject. A local coordinate system for the brim of the hat was constructed from the rigid body markers using their relative location in the global coordinate frame of the VICON camera system. A temporary marker was placed at the point where the laser beam was emitted from the laser pointer (P_1_), and the position of laser emission determined within the hat’s local coordinate frame. The projection of the laser beam onto the wall (P_0_) during this static calibration trial was also determined within the local coordinate system of the hat by placing a reflective marker at the laser’s point of intersection with the wall. The markers indicating the laser emission point and its intersection with the wall were removed for the dynamic trials. These positions could then be reconstructed during dynamic trials from their positions in the local coordinate system of the hat on the static trial, and used to determine the unit vector (û) along the line of projection of the beam. In addition, three static reflective markers (W_1_, W_2_, W_3_) were placed on the wall during the static calibration trial to determine the rigid wall’s position in the global calibration system. Then, the point of laser emission and the vector representing the beam’s projection direction was reconstructed from the hat local coordinate system at each sample of the dynamic trials. Along with the AP distance of the hat to the wall (W), these points were used to determine the location of the beam’s instantaneous point of intersection with the wall during dynamic trials during post-processing.

Body segment lengths were calculated between the adjacent joint markers. The reflective markers coordinates at each data sample were used to calculate the following sagittal plane joint angles (θ_i_): ankle, knee, hip, between lumbar 5^th^ and sacral 1^st^ vertebrae (L5-S1), between cervical 7^th^ and thoracic 1^st^ vertebrae (C7), and between the atlas and occipital condyles (AO) using a link-segment model [20]. The positive angle was defined as the upper (or cranial) segment moving anterior, based on the formula:


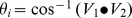


where V_1_ and V_2_ are unit vectors for the proximal and distal segments, respectively.

The location of the body center of mass (CM_POS_) was calculated by the sum of the product of each segment’s estimated mass and the location of center of mass of each segment, divided by total body mass. The estimated location of each segment’s center of mass was calculated from marker data, and the contribution of their masses to total body mass was estimated from Winter [20].


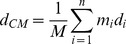


CM_POS_ was estimated using both a six segment (i.e., foot plus shank, thigh, pelvis, trunk plus arms, neck, head) and a four-segment (i.e., foot plus shank, thigh, pelvis, head-arms-trunk [HAT]) model.

### Experimental Measures

#### Targeting Error

Constant error and variable error with respect to the moving cursor were computed during the tracking movement to evaluate the accuracy and consistency of performing the tracking task.

Constant error estimated performance accuracy as follows:


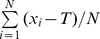


where *T* is the target position, *N* is the number of frames and *x_i_* is the position of the laser’s projection on the screen on the *i^th^* cycle.

Variable error estimated the consistency of targeting as follows:


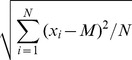


where *M* is the average position of the laser’s projection on the screen across frames [21].

#### Components of Joint Configuration Variance

UCM analysis was performed at each point in normalized time, across all tracking cycles, to determine the extent to which the variance of six sagittal plane joint motions led to variability of the presumed performance variables, head orientation to the target (HEAD_ORI_) and center of mass position (CM_POS_), or reflected equivalent joint configurations that stabilized the values of those performance variables [22].

UCM analysis can be illustrated conceptually as follows. Consider placing your index fingertip on a spot on your cheek to stop the bleeding after cutting yourself while shaving. This requires the control of the fingertip in three dimensions, X, Y and Z because orientation of the finger is not crucial to place the fingertip on the correct spot. Thus, the task requires three DOFs of fingertip control. However, the combined scapula, arm and finger have a total of at least thirteen joint angles that can be combined in different ways to achieve the same fingertip position, and more if we assume trunk motion. The system of joints is highly redundant with respect to the task’s requirements. This allows for flexibility in combining the joints across repetitions to place the fingertip on the spot, or across time to keep the fingertip on the spot. For example, if a fly lands on your elbow while maintaining pressure on the spot, there are enough redundant DOFs (13−3 = 10) to flick the elbow and chase the fly away. This has led to its characterization as motor abundance [23]. Across repetitions or across time, variations of the joint angles could lead to the finger leaving the designated spot on the cheek or could have no effect on the fingertip position. That is, if we consider the full space of the joints angles (N = 13), a subspace exists within which changes in the joint configuration have no effect on the selected fingertip position (13–3 = 10) and variations within this subspace allow for flexibility in performance. This is referred to as the UCM subspace and its linear estimate is the null space of the geometric model relating small changes in joint angles to changes in the performance variable of interest, here the fingertip position. However, the complementary subspace that lies orthogonal to the UCM subspace, mathematically, is the range space. Variations of the configuration of joint angles within this subspace leads to a range of fingertip positions that differ from the desired position. UCM analysis provides a formal way to determine how much of joint variance lies within the UCM (V_UCM_) versus the range space (V_ORT_). If you try to maintain a fixed position of your fingertip across time, or achieve the same position across repetitions of reaching to that spot, by using an identical joint configuration each time, then to the extent you are successful, we can expect that V_UCM_ ≈ V_ORT_. To the extent that you are not very good at replicating the spot, we can expect V_ORT_ > V_UCM_. There is no a priori reason to expect V_UCM_ > V_ORT_. Such a result would reflect a system that tends to make use of the available redundancy where possible to allow for flexibility, e.g., so that multiple tasks (keeping the finger on the spot while flicking that fly) can be performed without disturbing each other. As discussed below, however, some of V_UCM_ can derive from a source other than coordination among the joint motions. We introduce a method here to distinguish between these two sources.

The analysis of CM_POS_ focused on stability in the anterior-posterior (AP) direction because the amplitude of sway in the AP direction on a normal surface is substantially higher than in the medial-lateral (ML) direction during standing [3], as was the case for the tasks studied here. HEAD_ORI_ was defined as the angle between 1) a vector defined between individual markers placed on the right mastoid process and immediately lateral to the right eye and 2) a vector from the right mastoid to the center of the moving cursor for the tracking task and to the midpoint between the two stationary targets for the quiet standing task.

Each cycle of tracking was identified based on the peak-to-peak of head position and divided into two half-cycles (upward and downward tracking). Each half-cycle was then extracted and time-normalized to 100 data points. To make the analysis of the two tasks more comparable, quiet standing trials also were divided into pseudo-cycles by choosing a duration equal to the average duration of cycles for each subject’s tracking task and then time-normalizing that period to 100 data points. UCM analysis then was performed across cycles of tracking or across pseudo-cycles of quiet standing separately at each normalized time sample, according to the following steps described with respect to CM_POS_. The method for testing hypotheses related to the head orientation is similar, except that the geometric model does not include segment masses. Details of this analysis can be found in Scholz and Schöner [22] or Krishnamoorthy et al. [24].

1. A geometric model relating the CM_POS_ to the joint angles (θ_i_), limb segment lengths (*l*_i_), relative segment masses (*m*_i_), and the distance of the individual segment masses from distal joint (*d_i_*) [20] was developed (Appendix S1) [12].
2. The Jacobian matrix (*J)*, composed of partial derivatives of this geometric model, was computed. The Jacobian describes how small changes in the joint angles (θ_i_) affect the CM_POS_.
3. The null space of this Jacobian was obtained using Matlab™, providing a linear approximation to the UCM, or the subspace in joint angle space within which changes in the joint configuration have no effect on the mean CM_POS_. The complementary subspace (subspace orthogonal to the UCM) or range space was also computed, representing a subspace in joint space in which changes in the joint configuration lead to changes in the CM_POS_. Both subspaces were estimated at each time-normalized sample based on the mean joint configuration (θ_mean_) across cycles at that sample (N = 67 per trial).
4. At each time-normalized sample, the difference between the current joint configuration θ_i_ and θ_mean_ was projected into the null-space (UCM) and the range space of *J*, producing scalar estimates of the extent to which the joint configuration was aligned with those two subspaces.
5. Variances of the projection lengths across cycles at each point in normalized time were computed within both the UCM and range spaces. The two variances then were normalized to the appropriate subspace dimension, *d_ORT_ = 1* for the range space related to control of the AP CM_POS_ and *d_UCM_ (n−d_ORT_) = 5* for the UCM subspace based on the 6-DOF geometric model and *d_UCM_ (n−d_ORT_) = 3* for the 4-DOF geometric model of the CM_POS_, yielding measures of variance per DOF, V_ORT_ and V_UCM_, respectively. Because sagittal plane head orientation is also a 1-dimensional performance variable, the variance components were normalized to the same number of dimensions as for CM_POS-6DOF_.
6. Because the variance components were relatively stable across the tracking cycle, we averaged each variance component across the cycle, yielding one measure of V_UCM_ and V_ORT_ for each performance variable of each condition for each subject.

What has been referred to as a UCM effect (i.e., V_UCM_ > V_ORT_) can result because a given performance variable is stabilized by coordinating joint motions within the UCM whenever there is variation of one or more of the joint angles to keep the performance variable constant. In addition, however, if the axis of a given joint’s motion lies nearly parallel to a dimension of the UCM subspace, then most of its variance will lie within the UCM geometrically and its effect on the performance variable will be minimal (i.e., minimal contribution to V_ORT_) even if its motion is not coordinated with the motions of other joints (e.g., consider the effect of forearm pronation and supination on the hand’s spatial position when the wrist is in a neutral position). To determine the extent to which identified UCM effects result from joint coordination, UCM analysis was repeated on the experimental data after removing covariation among the joints by eliminating the off-diagonal terms of their covariance matrix, simulating a lack of interjoint coordination [25]. The extent to which the UCM effect was diminished by removing joint motion covariation provided an indication of the extent to which interjoint coordination was responsible for identified UCM effects. To reduce the number of factors in this analysis, the relative difference between V_UCM_ and V_ORT_ was computed for all performance variables:


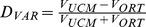


If D_VAR_ is close to ‘+1′, most joint variance reflects the use of motor abundance (i.e., many equivalent joint configurations) to stabilize the performance variable [22], [26]. To the extent that coordination among the joints accounted for UCM effects found in UCM analysis of the original data, removing joint covariation should result in D_VAR_ decreasing to near zero.

In addition, because of predicted differences in the coordination of cervical DOFs related to stabilizing the body in space (CM_POS_) versus producing a consistent head orientation, the individual contributions of each joint to D_VAR_ was determined for the CM_POS_ based on the 6-DOF model and for HEAD_ORI_. This can be accomplished by adding an additional step between steps #4 and #5 above. Because the UCM space and range space are subspaces of the original joint configuration space, the length of projections of each mean-free joint configuration into those two subspaces can be projected back into the full joint space to yield the proportion of the UCM and range space vectors contributed by each joint. Thus, for each joint there will be two contributions, one to the UCM subspace and one to the range space. The variances of each joint’s contribution to each subspace are then computed. D_VAR_ was then calculated based for each angle’s contribution to V_UCM_ and V_ORT_.

Finally, because many postural studies assume that control of only the ankle and hip joints are crucial for understanding upright posture [4], [6], [7], [27], including the performance of tracking tasks with the head [9]–[11], a 2-DOF geometric model of CM_POS_ also was evaluated. Moreover, several recent accounts of postural control have assumed, at least implicitly, that the nervous system cares more about the orientation of the leg and trunk segments in space than positional control of the ankle or hip joints [8], [28]–[31]. Therefore, UCM effects were computed based on a two-segment model of the CM position, using the angles formed by the leg (vector from the lateral malleolus to the hip joint) and the trunk (vector from the hip joint to the 7^th^ cervical marker), respectively, with the horizontal. Masses and mass locations of the shank plus thigh and the combined pelvis, head, arms and trunk were used to estimate the overall CM of the body in the model.

### Statistics

A repeated-measure analysis of variance (RM-ANOVA) first was performed to determine if differences existed in the variance components between the upward and downward phases of tracking, as well as between the half-cycle times for these two phases.

To investigate differences in the range of joint excursion between the tasks, quiet standing and tracking the moving target, a multivariate repeated-measure ANOVA was performed with task as the repeated factor. Which joints accounted for a significant multivariate effect were determined by the univariate ANOVAs.

A two-way repeated-measure ANOVA was performed for each performance variable (i.e., CM_POS-6DOF_, CM_POS-4DOF_ and HEAD_ORI_) to test for effects of the task (quiet standing vs. tracking) and differences between the variance components (V_UCM_ vs. V_ORT_). Significant interactions or planed contrasts were investigated using the m-matrix structure in SPSS™ version 18.0, with acceptable p-value of 0.05 for all analysis.

To compare the effect of actual and simulated UCM results (obtained after removing joint covariation), a RM-ANOVA was performed on D_VAR_ for all performance variables. In addition, a multivariate RM-ANOVA was performed to evaluate the contributions of each individual joint to D_VAR_ related to HEAD_ORI_ and CM_POS-6DOF_ before and after removing covariation among the joints.

## Results

### Cycle Time

The cycle time for the tracking task, based on the head’s movement, was 3.853±0.212 seconds. The half-cycle times did not differ significantly (p = 0.343) between the upward (1.924±0.19 sec) and downward (1.93±0.187 sec) phases of tracking.

### Range of Joint Motion

**Figure 2:**
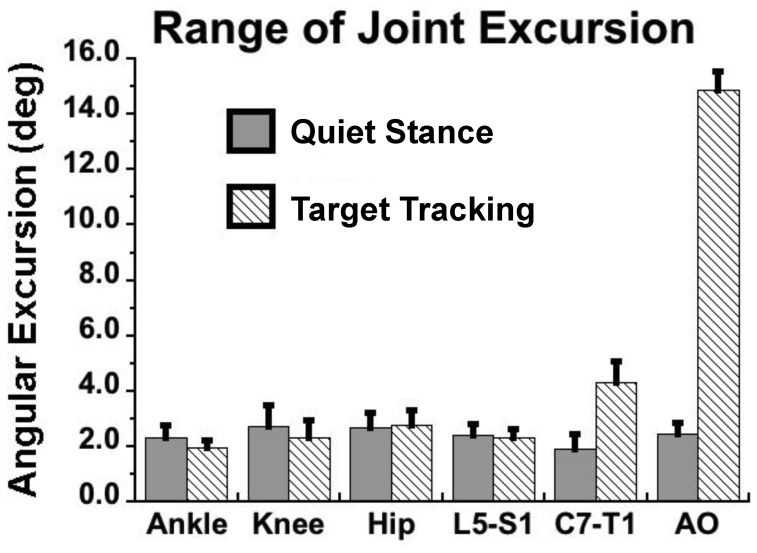
Range of joint excursion. Mean across subjects (±SEM) of the maximum joint excursions for all measured joint motions for quiet standing while visually fixating a point between two stationary targets and across cycles of tracking the moving target with the head; L5-S1 =  joint between 5^th^ lumbar and 1^st^ sacral vertebrae, C7-T1 =  joint between 7^th^ cervical and 1^st^ thoracic vertebrae. Cycles were defined as pseudo-cycles for quiet standing with the same average cycle time as with target tracking.

A multivariate repeated measures ANOVA revealed a significant difference in the average within-cycle range of joint excursions between the quiet standing and head tracking tasks (F_6,6_ = 80.1, p<0.001). The univariate ANOVAs revealed that the range of motion of the AO (F_1,11_ = 260.8, p<0.001) and C7-T1 joints (F_1,11_ = 26.4, p<0.001) were higher during tracking than during quiet standing. In contrast, excursions of L5-S1 (p = 0.65), hip (p = 0.17), knee (p = 0.08) and ankle (p = 0.76) joints did not differ between tasks (Figure 2). Moreover, differences between the tasks in the magnitude of across-cycle joint variability (F_6,6_ = 38.1, p<0.001) was found only for the AO (F_1,11_ = 38.1, p<0.001) and C7-T1 joints (F_1,11_ = 10.6, p<0.01), but not the other joints (all joints p>0.27).

Given the measured range of ankle joint excursion (Figure 2), the target distance (2-m) and its size (5-cm), this amount of ankle sway alone would lead to constant errors of targeting of between 2.8-cm and 9.0-cm for the range of subject heights (1.58-m to 1.85-m) without compensation by motion of other joints of the body.

### Performance Accuracy

**Figure 3:**
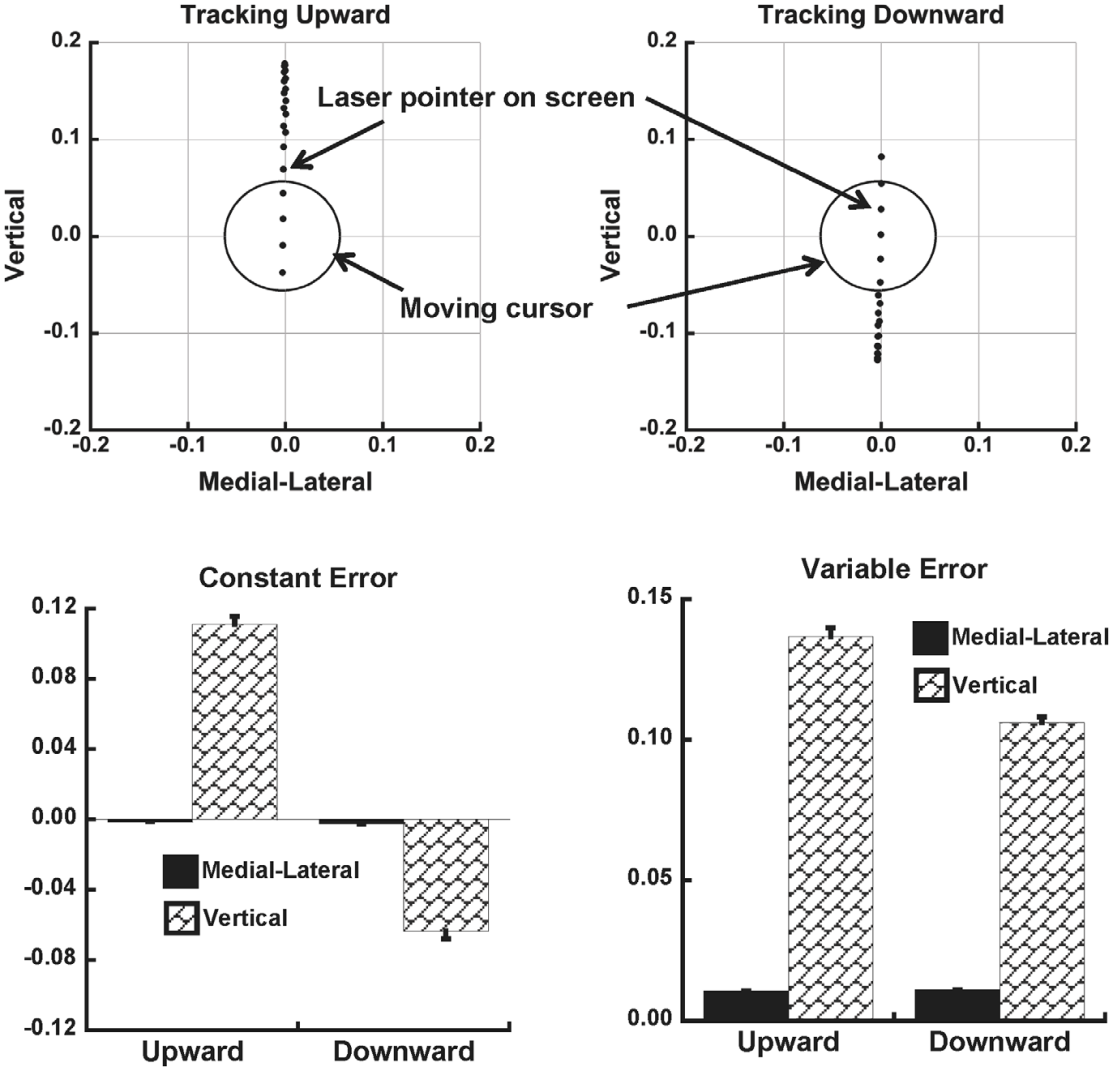
Constant and variable error of targeting. Upper Panels: Average across trials projection of the laser pointer (black dots) onto the screen at every 10 frames of tracking for one representative subject, after each cycle was normalized to 100 frames. Units are in meters (m). Lower Panels: Average across-subjects constant error (±SEM) and variable error (±SEM) of targeting with respect to the moving cursor are shown.

Accuracy and consistency of tracking performance was evaluated by computing constant and variable errors of targeting [21]. These errors were computed for each half-cycle of tracking (i.e., upward and downward). The number of cycles was consistent across subjects (65-cycles for each trial). Figure 3 shows the average across-subjects constant error and variable error in the medial-lateral (ML) and vertical directions. Constant error was substantially higher in the vertical direction during both upward (11.11±0.48-cm) and downward (−6.35±0.47-cm) tracking compared to the ML direction (upward  =  −0.12±0.008-cm; downward  =  −0.25±0.1-cm). The same was true for variable error (upward: Vertical = 13.67±0.31-cm; ML = 1.07±0.04-cm; downward: Vertical = 10.62±0.2 cm; ML = 1.1±0.05-cm).

### Components of Joint Configuration Variance

No significant differences were found when evaluating the effect of upward versus downward tracking movements on the variance components (F_1,11_ = 0.398, p = 0.533), nor was there a significant interaction between direction and variance component (F_1,11_ = 0.804, p = 0.377). Therefore, we confine our presentation of the head tracking results to the upward phase of head tracking.

**Figure 4:**
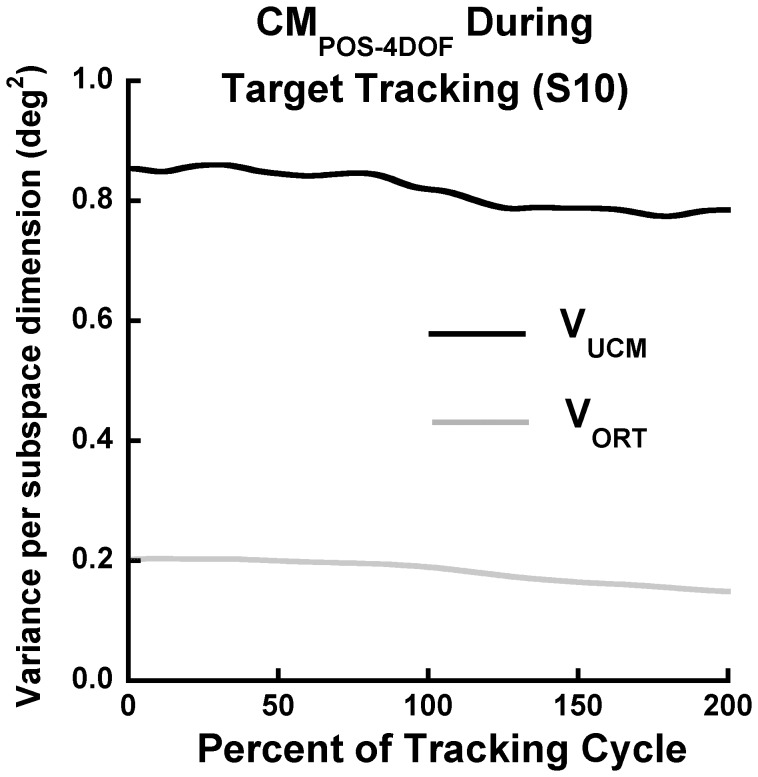
Example of components of joint configuration variance across the tracking cycle. Components of joint configuration variance per dimension of joint subspace (deg^2^) across the tracking cycle, related to stability of the 4-DOF CM position of a representative subject. Note the consistency of V_UCM_ and V_ORT_ across the cycle, leading to using the average across the cycle for further statistical analyses.

As noted, the UCM analyses were performed across multiple cycles at each percentage of normalized time. Figure 4 illustrates the results for each variance component at each percentage of the cycle for the CM_POS-4DOF_ of one representative subject. The results were similarly consistent for all performance variables. Therefore, the remaining figures show the results after averaging across the normalized time points.

**Figure 5:**
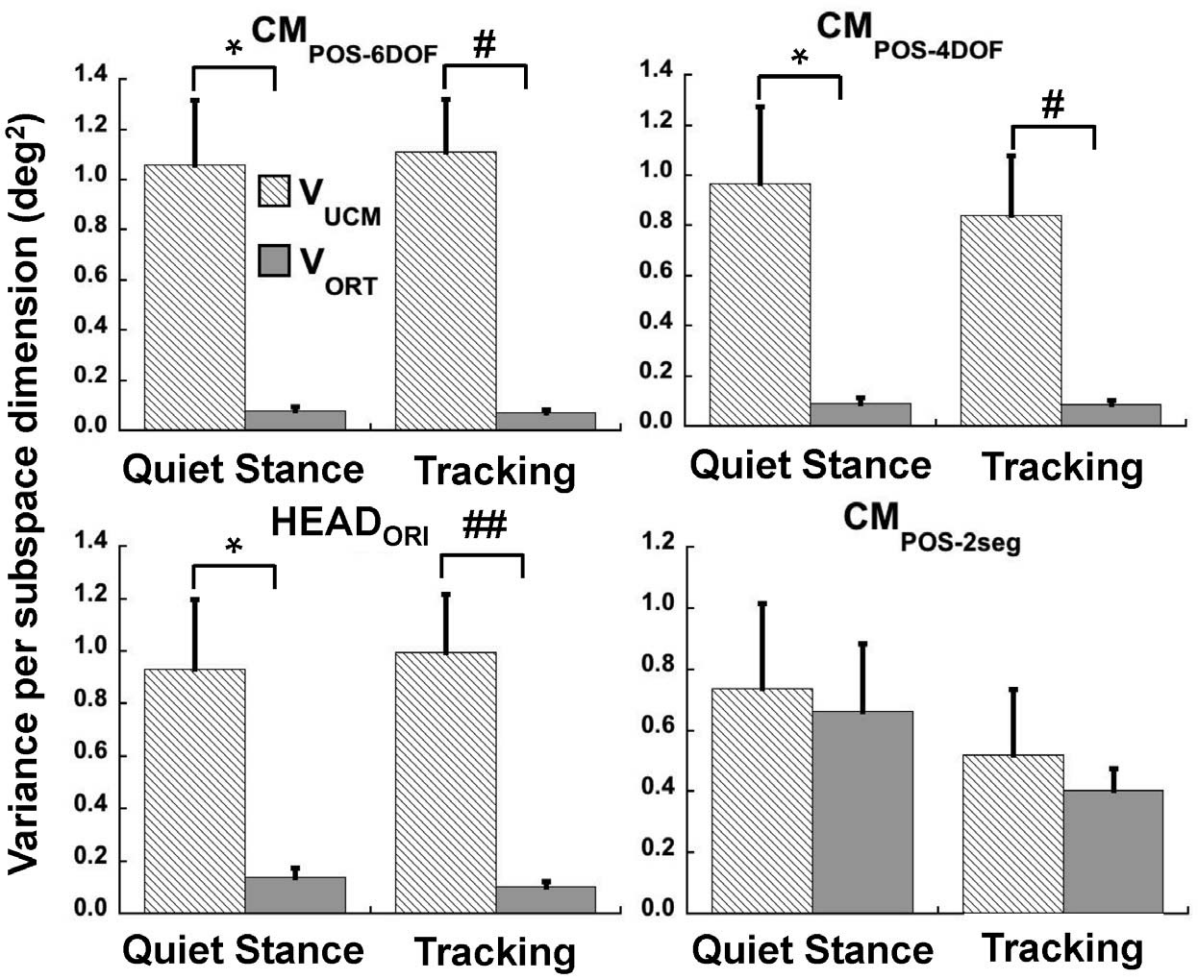
Average components of joint configuration variance. Mean (±SEM) components of joint configuration variance per dimension of joint subspace, related to stability of the center of mass position based on a 6-joint (CM_POS-6DOF_) and a 4-joint (CM_POS-4DOF_) geometric model and a 2 segment model, and for head orientation with the target (HEAD_ORI_), all computed during quiet standing while visually fixating a point between two stationary targets and across cycles of tracking the moving target with the head. Significant differences between V_UCM_ and V_ORT_: ^*^p<0.05; **^#^**p<0.005; **^##^**p<0.001.

In Figure 5, the average across-subjects components of joint configuration variance for quiet standing and the tracking task is presented for each performance variable. Strong UCM effects (i.e., V_UCM_ >> V_ORT_) were present for each performance variable for both the quiet standing and tracking tasks: CM_POS-6DOF_ (quiet standing: F_1,11_ = 153.1, p<0.001; tracking: F_1,11_ = 345.8, p<0.001; upper left panel, Fig. 4) and HEAD_ORI_ (quiet standing: F_1,11_ = 133.9, p<0.001; tracking: F_1,11_ = 78.3, p<0.001; lower left panel, Fig. 4). The results for center of mass position with the 4-DOF model (i.e., ankle, knee, hip and L5-S1 joints) also revealed large differences between V_UCM_ and V_ORT_ (Fig. 4, upper right panel; quiet standing: F_1,11_ = 91.0, p<0.001; tracking: F_1,11_ = 64.3, p<0.001). The UCM results differed significantly between the 6-DOF and 4-DOF models, reflected by a significant interaction of task variable and variance component for both conditions (quiet standing: F_1,11_ = 6.55, p<0.05; tracking: F_1,11_ = 10.4, p<0.01) in a separate RM-ANOVA. The interaction revealed in both cases that V_UCM_ was significantly greater when the model included the cervical joints, whereas V_ORT_ did not differ between the 4-DOF and 6-DOF models of CM_POS_.

There were no main effects of task (quiet standing vs. tracking; HEAD_ORI_: p>0.86; CM_POS-6DOF_: p>0.82; CM_POS-4DOF_: p>0.80), nor were there any significant interactions between the tasks and the variance components (HEAD_ORI_: p>0.35; CM_POS-4DOF_: p>0.42). However, the interaction approached significance for CM_POS-6DOF_ (F_1,11_ = 4.3, p = 0.06) as a result of slightly higher V_UCM_ and slightly lower V_ORT_ for tracking compared to quiet standing (Figure 4, lower left panel).

Results of the UCM analysis for the CM_POS_ based on a two-segment model are presented in the lower right panel of Figure 4. There were no significant differences between V_UCM_ and V_ORT_ (p>0.51); there was also no effect of task (p>0.09) or interaction between task and variance component (p>0.78).

### Components of Joint Configuration Variance after Removing Joint Covariation

**Figure 6:**
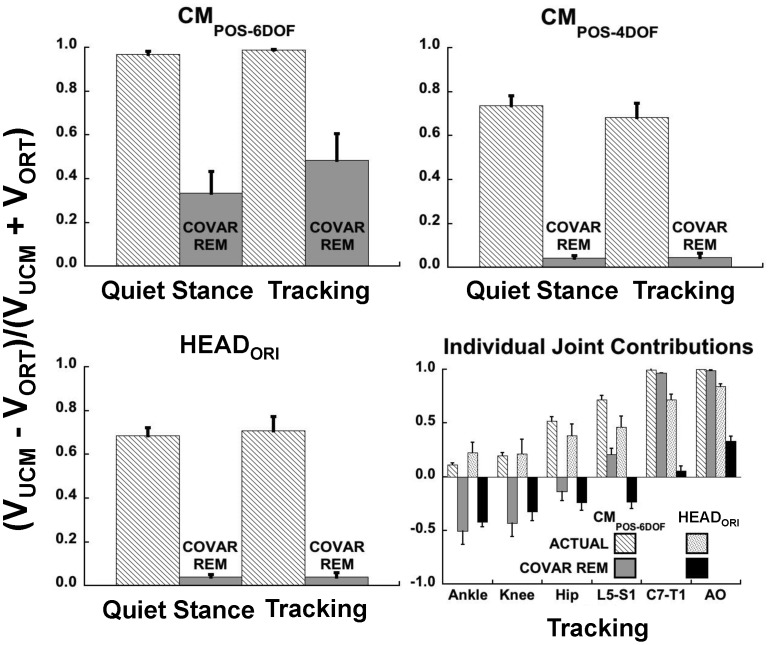
Relative difference between components of joint configuration variance, overall and for individual joints. Mean (±SEM) normalized variance difference between V_UCM_ and V_ORT_ for quiet standing and target tracking tasks before (patterned bars) and after (solid bars) removal of covariance among the joints (COVAR REM) by randomization, displayed for each performance variable. Values close to 1.0 indicate that most of the joint variance was V_UCM_. The amount of decrease after removing joint covariance reflects the extent to which V_UCM_ was due to multijoint coordination. Possible values range between −1.0 and 1.0. Only the positive range is shown for the upper two panels and lower left panel because no values fell below zero, unlike individual joint contributions. Lower right panel shows individual joint contributions based on both the 6-DOF CM model and HEAD_ORI_ variables for the target tracking condition. Results for quiet standing differed minimally from those for target tracking.

Figure 6 presents the relative variance difference (D_VAR_) based on UCM analysis of the actual data and after simulated removal of covariation among the joints. Values of D_VAR_ above zero indicate that more of the joint variance was V_UCM_, i.e., variance that would not change the value of the performance variable. A value of D_VAR_ approaching unity indicates that most of the joint variance was V_UCM._ In addition, the more that D_VAR_ decreased after removing covariation among the joints, the stronger the evidence that V_UCM_ resulted from coordination among the joint motions as compared to variation of individual joints whose axes happened to lie close to a dimension of the UCM.

RM-ANOVAs revealed a significant effect of removing covariation (CM_POS-6DOF_: F_1,11_ = 22.3, p<0.01; CM_POS-4DOF_: F_1,11_ = 129.0, p<0.001; HEAD_ORI_: F_1,11_ = 142.3, p<0.001). Only for CM_POS-6DOF_ (upper left panel, Fig. 5) was there a significant effect of task (F_1,11_ = 13.2, p<0.01) or an interaction of task with method (i.e., normal or simulated analysis; F_1,11_ = 8.0, p<0.05). This was due to a greater reduction in D_VAR_ when covariation was removed in the quiet standing task compared to the tracking task. The pattern of change between the results on actual versus simulated data were identical for CM_POS-4DOF_ and HEAD_ORI_ (Wilcoxon signed rank test, p = 0.78), both performance variables showing a large decrease in D_VAR_ after removing joint covariation (Fig. 6, upper right and lower left panels). Moreover, this decrease was substantially greater than for CM_POS-6DOF_ (Wilcoxon signed rank, p<0.01 and p<0.05 for comparison with CM_POS-4DOF_ and HEAD_ORI_, respectively). For all performance variables, the decrease in D_VAR_ resulted both from a decrease in V_UCM_ (CM_POS-6DOF_: F_1,11_ = 6.0, p<0.05; CM_POS-4DOF_: F_1,11_ = 5.7, p<0.05; HEAD_ORI_: F_1,11_ = 15.1, p<0.01) and an increase in V_ORT_ (CM_POS-6DOF_: F_1,11_ = 6.0, p<0.05; CM_POS-4DOF_: F_1,11_ = 11.2, p<0.01; HEAD_ORI_: F_1,11_ = 15.6, p<0.01).

The lower right panel of Figure 6 shows the contribution to D_VAR_ of individual joints before (light patterned bars) and after (dark filled bars) removing joint covariation for the HEAD_ORI_ and CM_POS-6DOF_ performance variables. For the ankle, knee, hip and lumbar spine, D_VAR_ decreased substantially after removing covariation. A multivariate RM-ANOVA revealed no differences between the tasks (i.e., tracking vs. quiet standing, p>0.63) or interaction (p>0.79) between the task and method (i.e., normal vs. simulated data). Therefore, further analyses of differences between individual contributions to HEAD_ORI_ vs. CM_POS-6DOF_ by method were examined for the tracking task only. For the ankle (p>0.70), knee (p>0.28) and hip (p>0.63) joints, the decrease in D_VAR_ after removing covariation did not differ between CM_POS-6DOF_ and HEAD_ORI_. In all cases, D_VAR_ became negative, indicating V_ORT_ > V_UCM_ without joint covariation. A similar decrease in D_VAR_ after removing covariation occurred for the L5-S1 joint, although there was a significant difference in the values related to stability of HEAD_ORI_ versus CM_POS-6DOF_ (Fig. 5, lower right panel; F_1,11_ = 14.2, p<0.01). D_VAR_ related to CM_POS-6DOF_ stability decreased significantly when removing covariation but it remained positive. In contrast, D_VAR_ related to the consistency of the head’s orientation was negative. On the other hand, differences in D_VAR_ before and after covariation removal between HEAD_ORI_ and CM_POS-6DOF_ for the cervical joints (C7-T1: F_1,11_ = 119.3, p<0.001 and AO: F_1,11_ = 156.2, p<0.001) were substantial. Although the cervical contributions to D_VAR_ did decrease slightly after removing joint covariation related to stability of CM_POS-6DOF_, this decrease was miniscule compared to the decrease related to HEAD_ORI_.

## Discussion

Results of the current study provide insights about the coordination of multiple joints along the body axis in providing for postural stability as well as consistency of the additional tracking task. Both single and double inverted pendulum models of posture have influenced strongly studies of postural control [3], [7], [8], [28], [32]–[36]. Recent work indicates, however, that both inverted pendulum models may be oversimplified [12], [24], [27], [37]. For example, if the body were controlled as a single inverted pendulum, all joint variance could be expected to be V_ORT_ because movement of the ankle would produce corresponding motion of the CM since that model presumes that all joints proximal to the ankle are stiffened and move minimally during quiet standing. If the body acted like a double inverted pendulum, involving motion about the ankle and hip, V_UCM_ could be higher than V_ORT_ only if the joint motions were perfectly out of phase. In such a case, the primary contribution of both ankle and hip joint variance could be expected to be to V_UCM_. However, out of phase motion about the ankle and hip appears to be limited to frequencies of sway above 2.0-Hz [8]. Moreover, results of the current study as well as previous analyses [38] indicate that, depending on the context, ankle joint variance contributes relatively equally to V_UCM_ and V_ORT_, with a tendency for a larger contribution to V_ORT_. Thus, a multi-DOF postural model based on the UCM hypothesis provides a viable alternative framework for understanding postural control [39]. The current results support that contention while revealing the role of coordination among the active DOFs in producing the experimentally identified variance structure related to stability of the CM_POS_ and consistency of head orientation. In addition, the results suggest that different synergies using some shared DOFs are used to stabilize the different performance variables.

### Is CM_POS_ Stability Dependent on Coordination of Cervical Joint Motions with those of more Caudal Joints?

The difference between UCM analyses performed with 4-DOF vs. 6-DOF geometric models of the CM_POS_ was the inclusion of cervical joint movements, including the AO joint. Indeed, the only significant increase in joint excursion when tracking an object in the current study, compared to the quiet standing condition, was due to cervical joint motion (Fig. 2). Studies of anticipatory postural adjustments (APAs) that have investigated muscle or joint synergies underlying postural perturbations or tasks with increased difficulty have shown, in general, similar differences in variance components (i.e., V_UCM_ > V_ORT_) related to stabilizing upright posture as those reported here [40]–[43]. Danna-Dos-Santos et al. [40] suggested that synergies of leg and trunk muscles underlying APAs help to stabilize the head, which also is important to ensure stable visual and vestibular information about the body’s position in space. Thus, it is somewhat surprising that adding the tracking task to quiet standing in the current study resulted in no substantial differences in either variance component across tasks. It may be that having subjects fixate on a stationary target during quiet standing, which required controlled head orientation to the fixation point, minimized differences compared to orienting to a moving target, although one would think that the added cervical and head motion in the latter case would affect postural stability.

The magnitude of the UCM effect (i.e., V_UCM_ > V_ORT_) was larger for the 6-DOF model of CM positional stability than when investigating the 4-DOF geometric model, due primarily to a greater magnitude of V_UCM_. V_ORT_ did not differ significantly between the two models (Fig. 4). This difference in V_UCM_ between the two models could be due largely to the fact that the axes of cervical joint motions were closely aligned with dimensions of the UCM. To distinguish between contributions to V_UCM_ due to inter-joint coordination versus body geometry, covariation among joints was artificially removed from the actual data [18], [19], [25] and the UCM analysis repeated. If joint covariation plays a dominant role, then most if not all of UCM effects found in the actual data set should disappear after removing it and repeating the UCM analysis. Verrell et al. [44] applied this randomization method to investigate the control of the CM position relative to the foot position at the time of heel strike during walking.

In the current study, when removing joint covariation from the 6-DOF geometric model there was still a significant UCM effect (V_UCM_ > V_ORT_). In contrast, the UCM effect largely disappeared when removing joint covariation from the 4-DOF model. This was because the values of the partial derivatives of the C7-T1 and AO joints with respect to the AP CM_POS_ in the Jacobian were an order of magnitude smaller than the values for more caudal joints (e.g., Ankle: 0.271; Knee: 0.265; Hip: 0.226; L5-T1∶0.178; C7-T1∶0.039; AO: 0.023 m/rad). Therefore, motion of the cervical joints could affect only minimally the CM_POS_. This fact is further emphasized when examining the contributions that individual joint’s motions made to the differences between variance components (Fig. 5, bottom right panel). For cervical joints, removing their covariation with other joints led to a miniscule reduction in D_VAR_ when considered with respect to the CM_POS-6DOF_.

These results lead to the conclusion that cervical joint motion was largely decoupled from the synergy that stabilized the AP CM_POS_ in the current task. It is conceivable that more vigorous head movements, perhaps associated with tracking at a higher frequency or larger amplitude, or tracking with the head in the horizontal plane, could lead to the need to coordinate cervical joint motions with those of more caudal joints to stabilize upright posture. The effect of these factors on the synergies identified here might be interesting to investigate in future work, but were beyond the scope of the current study.

### Is Orienting the Head to a Moving Object Accomplished by Independent Control of Cervical Joint Motions?

For the tallest subject of this study (1.85-m), tracking a 5-cm diameter target at a distance of 2-m, considering postural sway about the ankle of 2–3°, could lead to approximately a 10-cm vertical deviation of the laser pointer. Thus, in principal, coordinating the motions of joints most related to the body’s position in space with cervical joint motion should be important for accurate tracking performance. Nevertheless, the constant error of aiming at the target was, on average, greater than 10-cm in the upward direction. This amount of error could have resulted because subjects did not coordinate their body sway with cervical joint motion.

Alternatively, this degree of error could have resulted despite attempts to coordinate cervical and more caudal joint motions because of the inherent difficulty of tracking a target with a head-mounted laser. That is, larger errors in the vertical direction may have resulted because individuals typically track moving objects primarily with eye motion, predicting the object’s future location [45]–[47], unless the object’s excursion is too great. Because the laser was mounted on the brim of a baseball cap, above eye level, attempting to track the moving cursor with the eyes likely would result in a tendency for the laser’s projection to lead the moving target during its vertical ascent, although subjects were instructed to keep the laser on target throughout the movement. However, if this explanation were true, then one could expect a phase lag of the laser projection behind the moving cursor during the downward movement, which was not the case (upper right panel of Fig. 3). Instead, the laser’s projection tended to lead the target during the downward movements as well, albeit to a lesser extent. Thus, the consistent phase lead of the laser’s projection with respect to the target may have resulted from subjects’ inexperience precisely performing such tracking tasks primarily with head movement, as well as difficulty dealing with the inertia of the head, particularly at the turn around points, where the phase lead was found to be largest.

Evidence that cervical joint motion was coordinated with the motion of joints more directly linked to postural control comes from the strong UCM effect measured with respect to consistency of the head’s orientation to the target (Fig. 4). This was true both when subjects were tracking the moving target and when fixating a stationary point in space during quiet standing. More important is the fact that this effect was due largely to covariation among the joint motions as revealed by reduction of D_VAR_ to close to zero after removing joint covariation (Fig. 5). Although the decrease in D_VAR_ related to HEAD_ORI_ after removing covariation was smaller for cervical joints, and particularly for the AO joint, than for other more caudal joints, it was nonetheless substantial (Fig. 5, bottom right panel).

Results of this study suggest two synergies working in parallel while sharing some of the same resources. That is, motions of the four caudal joints are coordinated to stabilize the CM_POS_ during both quiet standing and the head-tracking task. This synergy apparently does not need to involve cervical joint motions because of their small effect on the CM_POS_. At the same time, consistent changes in the head’s orientation needed to track the moving target were not limited to independent control of cervical joint motions because distal joint motions can affect that orientation. Instead, given the motion of the caudal joints related to CM_POS_ control, another synergy appears to operate to coordinate cervical joint motions with those caudal joint motions to achieve a stable path of head orientation. Thus, the UCM method allowed a distinction to be made between coordination of the same joints related to two different performance variables, stability of the CM position and consistency of head orientation to the target.

### Is the Study of Multijoint Coordination Along the Kinematic Chain Necessary to Understand Postural Control?

The vast majority of studies focus on understanding the control of only the hip and/or ankle joints to achieve an understanding of upright postural control [4], [27], [34], [48]–[50]. Other recent work has focused on control of the orientation of two body segments in space, the leg (movement around the ankle) and trunk (movement around the hip), in studying the sensory information used to guide upright posture [8], [28]–[31], [51]. In contrast, recent research has suggested that coordination of multiple joints along the kinematic chain of the body is required for postural stability [12], [24], [52]–[55]. A consideration of how multiple joints and muscles contribute to posture is believed to be critical for an understanding of how supra-postural tasks can be integrated with the control of posture [41], [53], [56]–[58]. The current study revisited this question by investigating UCM effects based on a 2-body segment geometric model that estimated the effect of changes in the leg’s or trunk’s spatial orientation on the AP CM position.

Results of the 2-body segment analysis revealed no differences between the variance component that leads to CM variability (V_ORT_) and the variance component that reflects equivalent joint postures (V_UCM_) that produce an identical CM position. In other words, based on such a model, one would have to conclude that CM stability is not a goal of the postural control system despite suggestions to the contrary [59]. Moreover, this conclusion is at odds with the results obtained in this study when using 4-DOF and 6-DOF joint models.

The greater effect of removing joint covariance on V_ORT_ in the current analyses can be explained by a recently proposed model of postural control [39]. The model proposes a control law that stabilizes a performance variable such as the CM position by transforming sensory information that specifies unwanted changes in that variable into coordinated control signals to all joints that act to minimize deviations of the value of that variable from a desired value. Perturbations due to inherent noise, or self-generated by volitional movement, affect all joints. Therefore, their effect will be evidenced within both the UCM (the null space being a linear estimate of that subspace) and the range space of joint space. However, the proposed control law leads to strong resistance of the perturbation effects only in the range space as a result of these coordinated control signals. Thus, removing natural inter-joint covariation would be expected to result in a larger increase in V_ORT_ if multijoint coordination plays an important role in the control of upright posture.

## Supporting Information

Appendix S1 **Geometric model relating the joint configuration to the center of mass position.** The geometric model relating the joint configuration to the center of mass position (*d_CMPOS_*) in the sagittal plane (AP) was formulated in terms of ankle, knee, hip and L5-S1 joint angles, with the addition of C7-T1 and atlanto-occipital (AO) joint angles, or leg and trunk segment angles with the horizontal. The model with 6 joint angles (*θ_i_*) is provided here, including 6 limb segment lengths (*l_j_*), the proportion of total body mass for each of these segments (*m_j_*), and the distance of the individual segment masses from the disital end where the mass of that segment is concentrated (*d_j_*), where *i* = {*ankle, knee, hip, L5-S1, C7-T1 and AO*} joint angles, and *j*  =  {*shank, thigh, pelvis, trunk, neck, head*} segments [20].
(DOCX)
