# Supplementary material for: Functional Synergies Underlying Control of Upright Posture during Changes in Head Orientation
Source: PLoS One. 2012 Aug 1;7(8):e41583. doi: 10.1371/journal.pone.0041583 (PMC3411567; doi:10.1371/journal.pone.0041583)
Supplement: Appendix S1 — Geometric model relating the joint configuration to the center of mass position. The geometric model relating the joint configuration to the center of mass position (dCMPOS) in the sagittal plane (AP) was formulated in terms of ankle, knee, hip and L5-S1 joint angles, with the addition of C7-T1 and atlanto-occipital (AO) joint angles, or leg and trunk segment angles with the horizontal. The model with 6 joint angles (θi) is provided here, including 6 limb segment lengths (lj), the proportion of total body mass for each of these segments (mj), and the distance of the individual segment masses from the disital end where the mass of that segment is concentrated (dj), where i = {ankle, knee, hip, L5-S1, C7-T1 and AO} joint angles, and j = {shank, thigh, pelvis, trunk, neck, head} segments [20]. (DOCX) [file pone.0041583.s001.docx]

**Figure S1. APPENDIX**

The geometric model for the AP CM_POS-6DOF_ is:

*d_CMpos_ = m_shank_* * [ *d_shank_* * *l_shank_* * cos(*θ_ankle_*) ] + *m_thigh_* * [ *l_shank_* * cos(*θ_ankle_*) + *d_thigh_* * *l_thigh_* * cos(*θ_ankle_* + *θ_knee_*)] + *m_pelvis_* * [ *l_shank_* * cos(*θ_ankle_*) + *l_thigh_* * cos(*θ_ankle_* + *θ_knee_*) + *d_pelvis_** *l_pelvis_* * cos(*θ_ankle_* + *θ_knee_* + *θ_pelvis_* )] + *m_trunk_* * [ *l_shank_* * cos(*θ_ankle_*) + *l_thigh_* * cos(*θ_ankle_* + *θ_knee_*) + *l_thigh_* * cos(*θ_ankle_* + *θ_knee_* + *θ_pelvis_* ) + *d_trunk_** *l_trunk_* * cos(*θ_ankle_* + *θ_knee_* + *θ_pelvis_* + *θ_L5-S1_*)] + *m_neck_* * [ *l_shank_* * cos(*θ_ankle_*) + *l_thigh_* * cos(*θ_ankle_* + *θ_knee_*) + *l_thigh_* * cos(*θ_ankle_* + *θ_knee_* + *θ_pelvis_* ) + *l_trunk_* * cos(*θ_ankle_* + *θ_knee_* + *θ_pelvis_* + *θ_L5-S1_*) + *d_neck_* * *l_neck_* * cos(*θ_ankle_* + *θ_knee_* + *θ_pelvis_* + *θ_L5-S1_* + *θ_C7-T1_*)] + *m_head_* * [ *l_shank_* * cos(*θ_ankle_*) + *l_thigh_* * cos(*θ_ankle_* + *θ_knee_*) + *l_thigh_* * cos(*θ_ankle_* + *θ_knee_* + *θ_pelvis_* ) + *l_trunk_* * cos(*θ_ankle_* + *θ_knee_* + *θ_pelvis_* + *θ_L5-S1_*) + *l_neck_* * cos(*θ_ankle_* + *θ_knee_* + *θ_pelvis_* + *θ_L5-S1_* + *θ_L5-S1_*) + *d_head_* * *l_head_* * cos(*θ_ankle_* + *θ_knee_* + *θ_pelvis_* + *θ_L5-S1_* + *θ_L5-S1_* + *θ_AO_*)]

The methods for testing hypotheses related to the other performance variables are similar, except that the geometric model does not include segment masses.
